# Properties of Eco-Friendly Cement Composites Made with Recycled Cement Mortar-Based Artificial Aggregates

**DOI:** 10.3390/ma18225115

**Published:** 2025-11-11

**Authors:** Katarzyna Kalinowska-Wichrowska, Edyta Pawluczuk, Krzysztof Granatyr, Małgorzata Franus, Marta Kosior-Kazberuk, Michał Bołtryk, Adam Masłoń

**Affiliations:** 1 Faculty of Civil Engineering and Environmental Sciences, Bialystok University of Technology, Wiejska 45A, 15-351 Bialystok, Poland; e.pawluczuk@pb.edu.pl (E.P.); krzysztof.granatyr@pb.edu.pl (K.G.); m.kosior@pb.edu.pl (M.K.-K.); m.boltryk@pb.edu.pl (M.B.); 2 Faculty of Civil Engineering and Architecture, Lublin University of Technology, ul. Nadbystrzycka 40, 20-618 Lublin, Poland; m.franus@pollub.pl; 3 Department of Environmental Engineering and Chemistry, Rzeszow University of Technology, al. Powstańców Warszawy 12, 35-959 Rzeszów, Poland; amaslon@prz.edu.pl

**Keywords:** artificial aggregate (AAs), recycled cement mortar (RCM), municipal waste incineration ash (MWIA), sediment from the bottom of a water reservoir (SBWR), metakaolin (MK), sustainable development, CO_2_ emission

## Abstract

Artificial aggregates (AAs) are man-made construction materials, and their properties greatly depend on their manufacturing process (e.g., granulation and hardening) and the raw materials used. The conducted research aimed to determine the most advantageous composition of artificial aggregates prepared based on three wastes simultaneously: municipal waste incineration ash (MWIA), sediment from the bottom of a water reservoir (SBWR), recycled cement mortar (RCM)- which was the main waste. A production process of such aggregates was also developed, with the setting of the hardening temperature (20 °C, 200 °C, 400 °C). The X-ray diffractometry (XRD), differential thermal analysis (DTA), and thermogravimetry analysis (TGA) were used to characterize the waste. Then, the properties of cementitious composites prepared with artificial aggregate with the best strength parameters of 0–100% of the natural aggregate were determined. Carbon footprint calculations were performed for the production of artificial aggregate, depending on its composition and for cementitious composites.

## 1. Introduction

It is estimated that the annual production of construction, agricultural, forestry, and industrial waste worldwide exceeds one billion tons and shows a growing trend [1]. Such tendencies reinforce the need to move towards circular resource loops and to close material cycles in the construction sector, as recently highlighted in current resource policy assessments [2]. The construction industry is one of the main sectors in Europe. Still, it is also responsible for the depletion of a significant amount of non-renewable mineral resources and approximately 30% of greenhouse gas emissions, mainly carbon dioxide [3,4]. Natural aggregates account for approximately one-third of all raw materials and resources consumed globally and, in quantitative terms, represent the largest group of extracted minerals [5]. Their annual production across the European Union, Russia, Turkey, the United Kingdom, and the member countries of the European Free Trade Association (EFTA) is estimated at 4.2 billion tons, which constitutes around 9.3% of global output. In contrast, Asian countries alone (including China and India) are responsible for nearly 70% of global aggregate consumption [6]. Approximately 30% of extracted aggregates are used in concrete production [7].

The inevitable depletion of natural resources over time and the growing awareness of sustainable waste management practices in developed countries have increasingly emphasised the importance of recycling and the reuse of waste materials, particularly those originating from construction and demolition activities [8,9]. One promising solution is the production of artificial aggregates from waste materials, which not only reduces the volume of landfilled waste but also enables the reintegration of secondary raw materials into the construction sector [10], as also demonstrated in recent LWA-by-waste studies [11]. The use of industrial, agricultural, or construction waste as an alternative to natural aggregate extraction contributes to environmental preservation and reduces the consumption of natural resources in line with the principles of sustainable development [12]. However, some solid wastes or industrial by-products cannot be directly used in concrete, as they may have a significant adverse impact on concrete properties and the environment. These materials may contain heavy metals, chlorides, or sulfates, which can be harmful to cementitious systems and pose risks to human health [13]. Such materials are often used as partial components in the production of lightweight artificial aggregates [14]. A lightweight aggregate (LWA) is a solid substance having a particle density of less than 2.0 g/cm^3^ and a loose bulk density of less than 1.2 g/cm^3^ according to EN 13055:2016 [15]. Artificial aggregates are obtained as a result of the granulation process, i.e., consolidation of solid particles in the size range of 1 to 500 mm into aggregates of larger sizes. Currently, the commonly applied granulation methods in producing AAs can be divided into agitation granulation and compaction granulation. Agitation granulation is a process that consolidates fine, moisturised particles into large pellets by tumbling them in a rotating drum or disc pelletizer, without the application of any external compacting force [10,16]. Compaction granulation is used to produce pellets of well-defined shape and size by a mechanical force [17,18]. After the compaction, the extruded fresh pellets were cut into a small lump (with 15 mm in length), and rolled to produce spherical granules that had a diameter of approximately 8–10 mm to avoid the corner effects of cylindrical pellets. After pelletization, it is important to adopt a suitable hardening approach to transform fresh pellets into solid aggregates. The classic hardening technique widely used in the production of artificial aggregates, is sintering [19,20]. Other technologies have also been developed, such as alkaline activation [21], cold bonding [22] or accelerated carbonation [23,24] as a sustainable alternative to hardening by sintering. The production of artificial aggregates most commonly involves the use of an alkaline activator (NaOH and Na_2_SiO_3_, either as a mixture or separately), lime, or Na_2_CO_3_ as a binding additive, which determines the type of manufacturing process—cold bonding [25], sintering [26], or autoclaving [27]. Artificial aggregates used in lightweight concrete typically result in lower mechanical properties compared to concretes with conventional aggregates, primarily due to the introduction of additional porosity [28], although recent infrastructure sector data indicate that such performance penalties can be context-specific and application-dependent [29].

In the study by Zhang and Gjorv [30], it was shown that a 1% reduction in concrete density led to an approximately 3% decrease in compressive strength. However, it is important to note that reduced density and increased porosity may be desirable in specific applications, particularly in the case of lightweight concrete. As demonstrated in the research by Wyrzykowski et al. [31], the use of lightweight concrete with a density of 1800 kg/m^3^, where 45% of the natural coarse aggregate was replaced with artificial aggregate, enabled the achievement of a net negative carbon emission of −290 kg CO_2_/m^3^, compared to reference concrete.

The conducted research aimed to determine the most advantageous composition of artificial aggregates prepared based on three wastes simultaneously (municipal waste incineration ash, sediment from the bottom of a water reservoir, and recycled cement mortar), with the main waste being RCM. A production process of such aggregates was also developed, with the setting of the hardening temperature (20 °C, 200 °C, 400 °C). The X-ray diffractometry (XRD), differential thermal analysis (DTA), thermogravimetry analysis (TG) and scanning electron microscopy (SEM) were used to characterize the microstructure of the artificial aggregate. Then, the influence of the presence of artificial aggregate on the properties of cement composites was determined. The produced artificial aggregates can be used in construction as an alternative to existing artificial aggregates, the production of which is energy-consuming and expensive.

## 2. Materials and Methods

### 2.1. Materials

CEM II B-V 32.5R cement was used for the tests, following the requirements of the standard [32]. Its basic parameters are presented in Table 1.

As natural aggregates (NA), the river sand fraction 0–2 mm and the gravel fraction 2–4 mm and 4–8 mm were used. Tap water meeting the requirements of EN 1008:2002 [33] was used for the preparation of artificial aggregate and subsequently for the fabrication of test specimens.

The following waste materials were used in the production of the artificial aggregate: municipal solid waste incineration (MSWI) ash from Bialystok (Poland), bottom sediment collected from the bed of a water reservoir near Rzeszów (Poland), and a fine recycled concrete fraction, which served as the primary component of the aggregate. This fraction, originating from the grinding of concrete floor slabs, in the study was referred to as recycled cement mortar (RCM)

Additionally, metakaolinite—obtained by calcining kaolinite at a temperature exceeding the dehydroxylation threshold—was used in the production of the artificial aggregate. Its purpose was to promote the formation of calcium silicate hydrate (C-S-H) and calcium aluminosilicate hydrate (C-A-S-H) phases within the artificial aggregate [34]. The chemical composition of waste raw materials and metakaolin used for the production of artificial aggregate is presented in Table 2.

Numerous studies confirm that there are significant differences in the elemental composition of fly ash and incineration ash [35,36]. As shown in the table, the examined ash is characterised by a high CaO content and low concentrations of SiO_2_ and Al_2_O_3_. Particular attention should be paid to the sulfur (SO_3_) content of 6.99% and chloride (Cl) content of 1.04%, which may induce concrete corrosion. Additionally, the incineration ash contained many visible, irregularly shaped, large particles, which are fragments of unburned municipal waste. This is evidenced by the very high loss on ignition (LOI = 23.33%).

Sediments should be chemically analysed before use to ensure that the material is not contaminated with potentially toxic substances [37]. The dominant component of the analysed sediment was SiO_2_, which accounted for 65.53% of its mass. Noticeable amounts of Al_2_O_3_, CaO, and Fe_2_O_3_ were also present. The composition of the reservoir bottom sediment can be considered as an indicator of water pollution.

In the recycled cement mortar (RCM), the predominant components were calcium and silicon oxides, accounting for 39.39% and 34.40% of the mass, respectively. The silica originated in part from the natural fine aggregate present in the recycled material.

**Figure 1:**
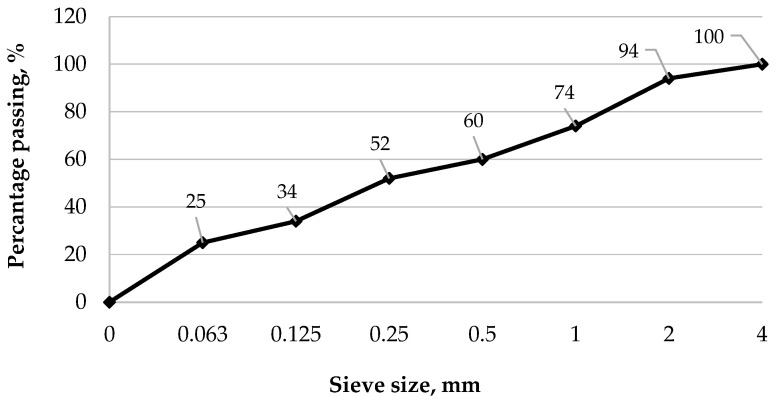
Grading curve of RCM.

The RCM sieve analysis was performed by EN 933-1:2012 [38], and the grading curve of RCM is presented in Figure 1.

**Figure 2:**
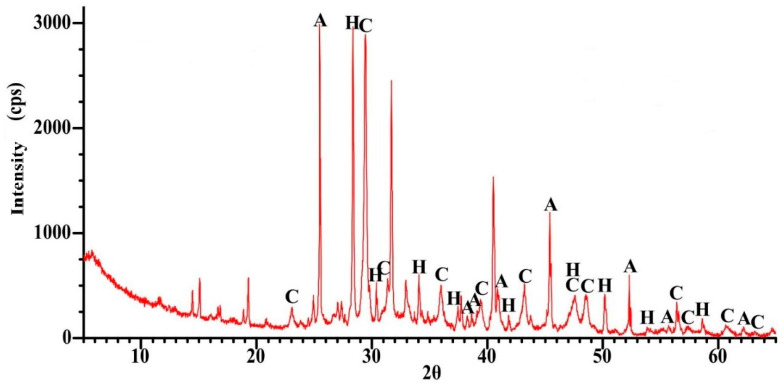
XRD pattern for MWIA, A—Anhydryte (CaSO_4_), H—Hibschite ((Ca_2.34_Fe_0.66_)(Al_1.856_Fe_0.144_)Si_2.853_O_11.412_(OH)_0.588_), C—Calcite (CaCO_3_).

**Figure 3:**
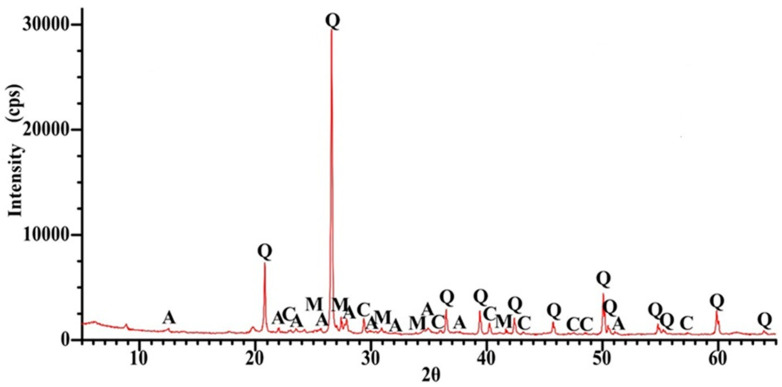
XRD pattern for SBWR, A—Anorthite (NaAl_2_Si_2_O_8_), M—Microcline (KAlSi_3_O_8_), Q—Quartz (SiO_2_), C—Calcite (CaCO_3_).

**Figure 4:**
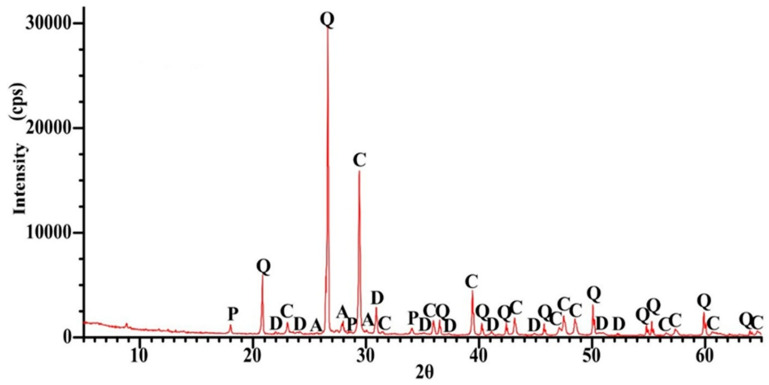
XRD pattern for RCM, A—Albite (NaAlSi_3_O_8_), Q—Quartz (SiO_2_), D—Dolomite (CaMg(CO_3_)_2_), C—Calcite (CaCO_3_), P—Portlandite (Ca(OH)_2_).

As shown in Figure 1, the dust fraction (<0.063 mm) in the recycled cement mortar (RCM) accounted for 25% of the total mass, while 74% of the material passed through a 1 mm sieve. For further analysis, the <0.5 mm fraction was selected, representing 60% of the entire material. The metakaolin used consisted primarily of alumina (Al_2_O_3_) and silica (SiO_2_), with a low loss on ignition (LOI) of 0.63%, which is consistent with the results reported in other studies [39,40]. Figure 2, Figure 3 and Figure 4 present the phase composition of the individual waste materials used in the production of artificial aggregate, as determined by X-ray diffraction (XRD) analysis.

**Figure 5:**
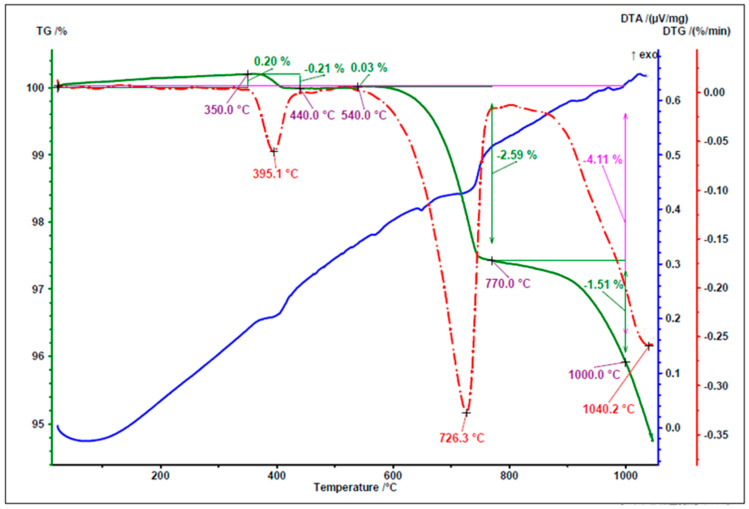
TG/DTG and DTA curves of MWIA.

**Figure 6:**
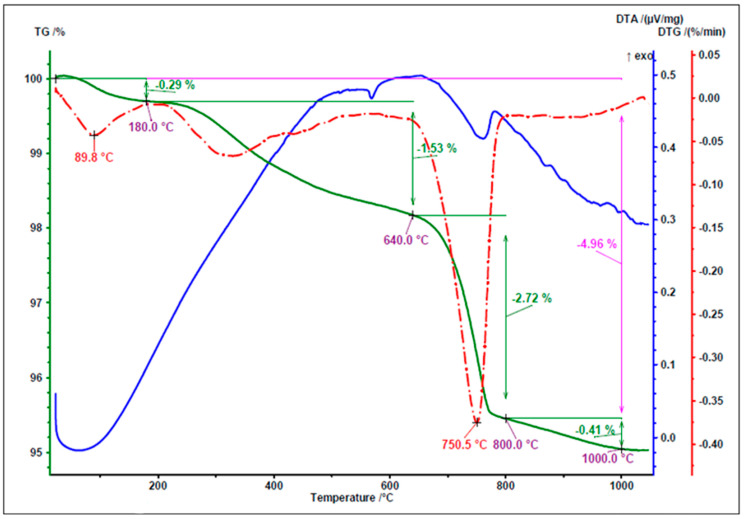
TG/DTG and DTA curves of SBWR.

**Figure 7:**
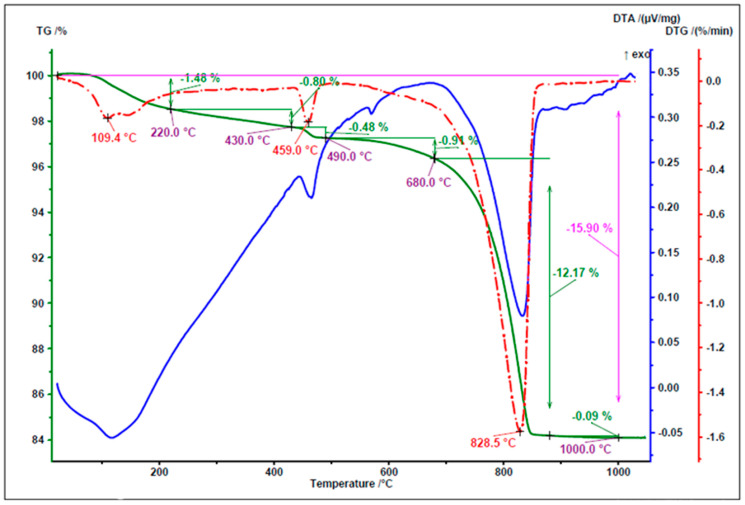
TG/DTG and DTA curves of RCM.

Figure 5, Figure 6 and Figure 7 present the results of thermal analysis of the waste materials used for the production of artificial aggregate (MWIA, SBWR, RCM) during the heating up to a temperature of 1000 °C.

For the analysed municipal solid waste incineration ash, the first significant mass loss occurred in the temperature range between 350 °C and 450 °C, with a distinct peak at 395.1 °C. A subsequent mass loss, associated with an exothermic reaction, was observed in the temperature range of 640–800 °C and was attributed to the decomposition of CaCO_3_, the presence of which was confirmed by XRD analysis (Figure 2).

In the thermal analysis, three temperature ranges can be distinguished. The first range up to 200 °C, in which a small mass loss of 0.29% occurred due to moisture loss. The next interval appeared between 200 °C and 640 °C, in which a mass loss of 1.53% occurred. It was probably related to the dihydroxylation of clay minerals (Anorthite and Microcline) detected in the XRD analysis (Figure 3). After that, the decomposition of CaCO_3_ takes place in the temperature range from 650 °C to 800 °C with a sharp peak at 750.5. The main reflection was found quartz (Figure 6), which is similar to the test results presented by other researchers [41].

Figure 7 shows three temperature ranges associated with the mass loss. Below 200 °C, moisture evaporation and ettringite dehydration occurred. At 459 °C, a peak was observed corresponding to the dehydration of portlandite (Ca(OH)_2_), while in the temperature range of 650 °C to 1000 °C, decarbonation of calcite was recorded.

Table 3 presents the content of bound water, portlandite, and calcite in RCM, calculated according to the method described in [42].

As shown in Table 3, the amount of calcium hydroxide present is relatively low, at 5.7%. In contrast, the high content of calcium carbonate observed in the experiment (27.83%) is a result of the fine grinding of the analysed material, which facilitated the progression of carbonation. This indicates that most of the Portlandite has already undergone carbonation. Part of the calcium carbonate also originates from the aggregate present in the recycled mortar. It is found that the polymerisation process necessitates a rapid reaction of silica (Si) and alumina (Al) in an alkaline environment, resulting in a three-dimensional polymeric chain of Si–O–Al–O linkages [43,44]. For this reason, an alkaline NaOH solution was used as the activator.

### 2.2. Research Methodology

The particle size distribution of the artificial aggregate was determined based on three samples, under EN 12620:2013 [45]. The bulk density of the artificial aggregate was established following the procedure outlined in EN 1097-3:2000 [46]. Water absorption of the aggregates was tested according to EN 1097-6:2022 [47]. The strength of artificial aggregates under compression was examined using the non-standard diametrical compression test, where the sample was placed on a stationary testing plate lengthwise. The experiment was carried out using a TA.XTplus Texture Analyser (Stable Micro Systems, Godalming, UK) equipped with a 0.5 kN load cell and a universal material testing machine Inspekt Table 50 kN (Hegewald und Peschke MPT GmbH, Nossen, Germany). The universal testing machine was used for strength investigations of granules requiring a force higher than 0.5 kN. The diversity of equipment used was selected due to the higher precision of TA.XTplus Texture Analyser is more accurate than the Inspekt Table, which allows for obtaining more accurate results for the weaker, in terms of strength, granules. The upper moving plate compressed granules of a measured height and diameter with a test speed of 2 mm·min^−1^ until the compression force dropped by 30%. Due to a low range of elastic deformations, the samples were destroyed each time. The test was repeated for min. 8 samples produced at each process condition. The compression stress (σ_n_) was calculated as follows:

(1) $$\sigma_{n}=\frac{2P}{{\pi d}^{2}}$$

where $\sigma_{n}$ is the aggregate strength, MPa, *P* is the load applied at failure, N; *d* is the average particle diameter of aggregates, mm.

**Figure 8:**
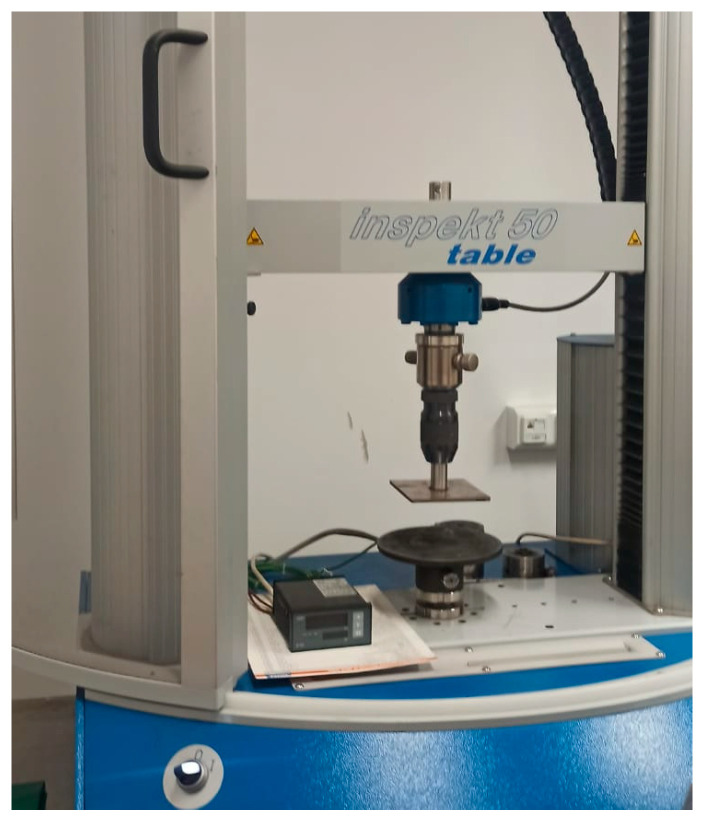
Apparatus for measuring the compressive strength of aggregates.

Figure 8 shows the apparatus used for measuring the compressive strength of aggregates.

The consistency of the mortar was tested on three samples in accordance with EN 1015-3:1999 [48]. The fresh mortar density was determined according to the guidelines of EN 1015-6:1998 [49], also based on three samples per series. Flexural and compressive strength tests of the composite were carried out in accordance with EN 196-1:2016 [50]. Specimens with dimensions of 40 × 40 × 160 mm were tested after 14 and 28 days of curing. The tests of water absorption were performed according to Polish standard PN-88/B-06250 Ordinary concrete [51] on 3 samples. First, the specimens with dimensions of 40 × 40 × 160 mm were dried in a laboratory oven at 110 °C to a constant mass and then weighed (m_1_). The prepared samples were subsequently immersed in water at a temperature of 18 ± 2 °C until fully saturated and weighed again (m_2_). The water absorption (WA) was determined using the following formula:

(2) $$WA=\frac{m_{2}-m_{1}}{m_{1}}\cdot 100\%,$$

To determine the chemical composition in the raw materials (MK, MWIA, SBWR, RCM), the X-ray fluorescence (XRF) was performed. XRF was performed using a 4 kW power source and ZSX PRIMUS IV (Rigaku Corporation, Tokyo, Japan) equipment. All the selected raw materials were characterised with XRD patterns. The XRD analysis was performed using a Bruker D8 Discover A25 instrument (Bruker AXS GmbH, Karlsruhe, Germany) with CuKα (λ = 1.54050 Å; 40 kV; 30 mA).was used. Diffraction patterns were measured between 10 to 70 (2θ) at a rate of 0.006 2θ min^−1^. The differential thermal analysis and thermogravimetric analysis were carried out using the NETZSCH analyser, model STA 409 PG (NETZSCH-Gerätebau GmbH, Selb, Germany), under an atmosphere of nitrogen. The specimens were heated at a rate of 10 °C/min to a temperature of 1100 °C. The morphology of artificial aggregates was investigated using a high-resolution SEM (TESCAN ORSAY HOLDING, Brno, Czech Republic; Aztek Automated by Oxford Instruments plc, Abingdon, UK) equipped with an X-ray microanalysis system (EDS) as well as a high-resolution FEI microscope (Quanta 250 FEG; FEI Company, Hillsboro, OR, USA), digitally controlled and equipped with a thermal field emission Schottky electron gun.

### 2.3. Production Process of Artificial Aggregate

**Figure 9:**
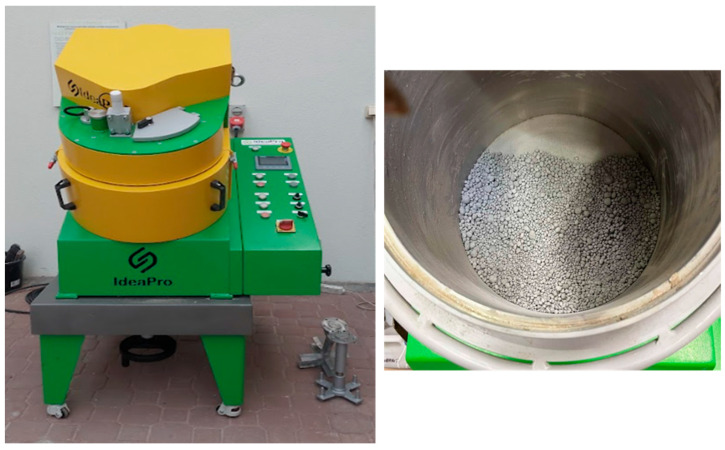
Stages of artificial aggregates production.

The production process of the artificial aggregate began with weighing the appropriate amounts of each component: RCM, MWIA, SBWR, MK, the activator, and water, as specified by the experimental design. First, the dry components were combined and mixed for 3 min. The dry mixture was then placed into a granulator. In the next step, the activator–water solution was gradually dosed by spraying, while the granulation process was continuously monitored (Figure 9).

The prepared artificial aggregate was kept under laboratory conditions for the first 24 h, after which it was subjected to curing at temperatures of 200 °C and 400 °C for 30 min. A portion of the aggregate was not thermally treated and was cured at 20 °C for a period of 28 days. After the cooling, the cured aggregate was sieved to obtain the 2–8 mm and 8–32 mm fractions, which were then used for further testing after the required curing period.

## 3. Test Results and Discussion

### 3.1. Composition and Properties of Artificial Aggregate

#### 3.1.1. Determination of the Type of Activator

**Figure 10:**
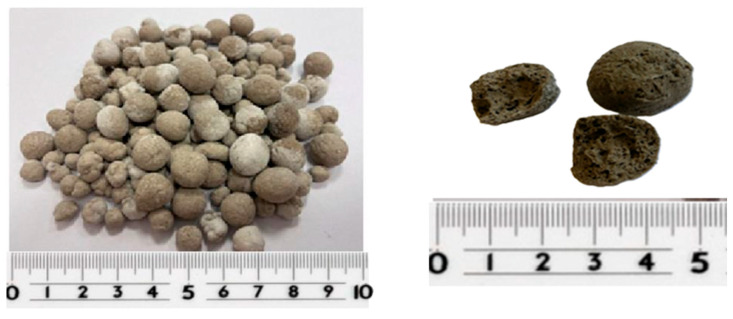
Ready artificial aggregates prepared for further tests (**left**) and cross-section of aggregates (**right**).

Before proceeding with the main part of the experiment, trial batches of artificial aggregate with various compositions were produced to determine the configuration yielding the highest strength of individual granules. The first step involved selecting the activator. For this purpose, trial aggregates were prepared using the following alkaline activators: 8 M NaOH, 10 M NaOH, and a sodium silicate solution (water glass). Based on the evaluation of the granulation process and the strength of the aggregates assessed under a defined load, 10 M NaOH was selected as the activator for further research. Figure 10 shows the 8–32 mm aggregate fraction designated for subsequent testing.

#### 3.1.2. Formulation of Aggregate Composition

To determine the optimal aggregate composition, several different mixture formulations were prepared, with observations focused on the granulation process and compressive strength. Based on these results, it was established that the addition of bottom sediment had a beneficial effect on the granulation process; therefore, it was included in the final aggregate composition for further research. The composition also incorporated municipal solid waste incineration ash, a particularly challenging waste material to manage. However, the primary waste considered for reuse remained recycled cement mortar (RCM), and its content was selected as one of the experimental variables. The second variable was the curing temperature of the artificial aggregate following the granulation process (Table 4).

Table 5 presents the compositions of the artificial aggregates, hereafter referred to as A, B, and C, while Table 6 shows the experimental design.

**Figure 11:**
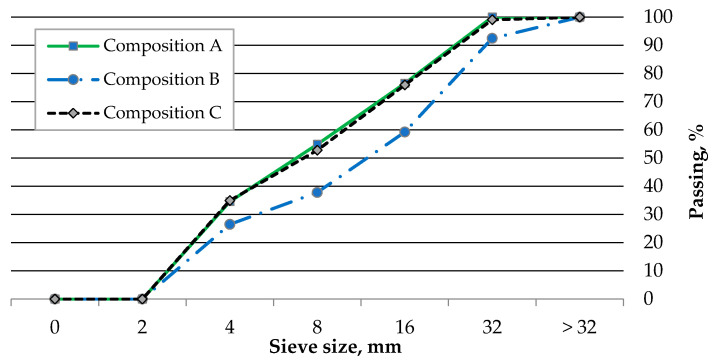
Sieve curves of artificial aggregate.

Figure 11 shows the particle size distribution curves of the individual aggregates, depending on their composition (A, B, C). The curves were obtained based on three samples for each composition.

As shown in the figure, composition B resulted in a lower proportion of fine aggregates (<4 mm) compared to compositions A and C, which exhibited similar particle size distributions. The content of the 8–32 mm fraction in composition B was 54%, while in compositions A and C it was 44% and 46%, respectively. After the artificial aggregates were produced and thermally treated, tests were carried out to determine loose and compacted bulk density, water absorption, and specific gravity. Additionally, a particle size distribution curve was prepared, and compressive strength measurements of individual aggregate particles were performed.

#### 3.1.3. Physical and Mechanical Properties of Artificial Aggregates

Table 7 presents the results of bulk density (loose and compacted), specific gravity, water absorption, and compressive strength tests for the individual aggregate compositions (A, B, C), depending on the curing temperature. Before the testing, the aggregates were divided into two size fractions: 2–8 mm and 8–32 mm. Compressive strength tests were performed on the 8–32 mm fraction.

With the increase in hardening temperature from 20 °C to 400 °C, a clear and gradual decrease in the bulk density of the artificial aggregates was observed. This trend was consistent across all compositions and both particle-size fractions. After the heating at 400 °C, the volume density of all aggregates decreased below 2.0 g/cm^3^, confirming the formation of a more porous internal structure. This structural change can be attributed to the removal of physically bound water and the onset of thermal decomposition processes in the waste-derived components.

At the same time, a marked increase in water absorption was recorded, particularly at the highest hardening temperature. At 400 °C, water absorption reached values in the range of 12.3% to 16.2% by mass. This pronounced rise is most likely the result of moisture evaporation and the partial combustion of organic particles present in the mixture—effects that are especially evident in the material containing municipal waste incineration ashes (MWIA). The increase in open porosity associated with these processes directly explains the simultaneous reduction in bulk and volume densities.

The compressive strength of the individual aggregate grains remained relatively low throughout the tests. The compressive strength of individual aggregate particles remained relatively low throughout the study period. Regardless of composition and curing temperature, the strength values generally did not fall below 0.4 MPa, but did not exceed 1.0 MPa as well. This confirms that, although the aggregates meet the density requirements for lightweight materials, they should be considered as the low-strength aggregates for structural applications.

Among the tested variants, the aggregates of composition B demonstrated the most favourable balance of properties. In particular, the material containing 50% recycled cement mortar (RCM) and 10% sediment from the bottom of a water reservoir (SBWR), after calcination at 400 °C, achieved the highest recorded compressive strength of 0.93 MPa. This strength, although still in the low range, is notably higher than that of the other compositions and is accompanied by acceptable density and absorption parameters. For this reason, aggregates of composition B calcined at 400 °C were selected for further testing as the most promising option for practical use in sustainable construction applications.

**Figure 12:**
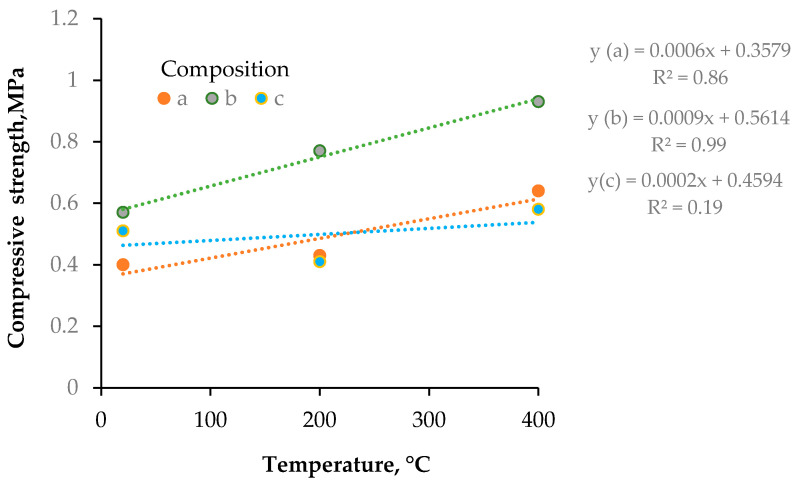
The correlation between the compressive strength and the temperature of heat curing of the aggregate.

In Figure 12, the correlation between compressive strength and the temperature of heat curing of aggregate was demonstrated.

Figure 12 illustrates the relationship between the compressive strength of the artificial aggregates and the temperature of heat curing for the three tested compositions (A, B and C). In all cases a slight overall increase in compressive strength is observed as the curing temperature rises from 20 °C to 400 °C, although the absolute values remain below 1 MPa. Composition B exhibits the most distinct positive trend, described by the regression equation (y = 0.0009x + 0.5614, R^2^ = 0.99); the compressive strength of this material rises from approximately 0.57 MPa at 20 °C to about 0.93 MPa at 400 °C. Composition A shows a more moderate increase (y = 0.0006x + 0.3579, R^2^ = 0.86), reaching roughly 0.64 MPa at the highest curing temperature. In contrast, composition C displays only a very weak temperature dependence (y = 0.0002x + 0.4594, R^2^ = 0.19), with compressive strength remaining close to 0.5 MPa across the investigated range. These results indicate that elevated curing temperature can enhance the mechanical properties of the aggregates. The significant effect is strongly dependent on the material composition and waste proportion in the aggregate mixtures.

### 3.2. Properties of Cement Composites with Artificial Aggregate

#### 3.2.1. Experimental Plan

Based on previous studies, the composition of the artificial aggregate intended for use in cementitious composites as a substitute for natural aggregate was established. The selection of the appropriate aggregate was based on the compressive strength tests and observations of the granulation process. The aggregate consisted of 80% solid materials and 20% alkaline activator with water, as specified in Table 5.

The testing of cementitious composites incorporating artificial aggregates was designed as an experiment involving two variables, as presented in Table 8. Due to the dimensions of the composite specimens (40 × 40 × 160 mm), the 2–8 mm fraction of artificial aggregate was selected for the tests

During the experiment, 10 series of specimens were prepared, including one control series (Series 10). The control series was made using 100% natural gravel aggregate with a particle size of 2–8 mm. Specimens with dimensions of 40 × 40 × 160 mm were produced. The tests were carried out after 14 and 28 days of curing under the laboratory conditions. The experimental plan is presented in Table 9.

In the experiment, a temperature of 20 °C was assigned to the series that were not subjected to thermal treatment but were stored under standard laboratory conditions.

#### 3.2.2. Cement Composite Mix Composition

Table 10 presents the designed composition of the composite mix depending on the percentage content of artificial aggregate.

The composite mix designs were prepared assuming the constant quantities of cement (320 kg/m^3^), water (160 kg/m^3^), and sand (878.6 kg/m^3^), with a fixed water-to-cement ratio (*w*/*c*) of 0.50.

The weighed components were added to the mixer in the following order: artificial aggregate (2–8 mm), natural aggregate (2–8 mm), sand, and cement, and mixed for approximately 1 min. Subsequently, water was added to the mixture, followed by 2 min of mixing. After this stage, the mixer walls were cleaned, and mixing was continued for an additional minute.

The fresh mix was cast into prismatic molds with dimensions of 40 × 40 × 160 mm. The specimens were compacted on a vibrating table for 1 min in two layers. After 24 h, the specimens were demolded and placed in water until reaching the designated curing ages of 14 and 28 days.

#### 3.2.3. Workability and Density of Fresh Cement Composite

**Figure 13:**
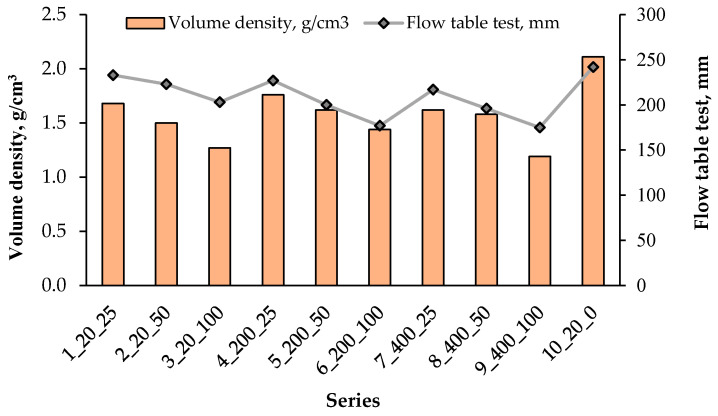
Relationship between the fresh mortar bulk density and its workability.

Consistency and fresh bulk density tests of the mortar were performed on three samples for each series, and the average values were calculated from the obtained results. The results are presented in Figure 13.

Figure 13 presents the results of the volume density measurements of the produced aggregates together with the corresponding flow table test values of mortars prepared with these aggregates. The volume density of the aggregates varies between approximately 1.2 and 2.1 g cm^−3^ across the tested series. In general, series produced at higher temperatures (200 °C and 400 °C) tend to lower volume density compared to those hardened at 20 °C, which reflects the formation of a more porous internal structure during thermal treatment. The lowest densities, close to 1.2 g cm^3^, are observed in series 3_20_100 and 9_400_100, while the highest density of about 2.1 g cm^3^ occurs in series 10_20_0, containing the reference aggregate without waste additions.

The flow table test results for the mortars range from roughly 150 to 250 mm. Although some fluctuations are visible, the general trend indicates that mortars containing aggregates of lower volume density tend to exhibit slightly reduced flow values, suggesting a moderate influence of aggregate porosity on the workability of the fresh mixture. The most fluid mixture (≈250 mm) corresponds to the reference series 10_20_0, whereas the lowest flow (≈150 mm) is found for series 6_200_100 and 9_400_100, both of which are characterised by relatively low aggregate density.

Overall, the combined data demonstrate that increasing the proportion of waste materials and the application of heat treatment promote the formation of lightweight aggregates, while simultaneously leading to a moderate decrease in the workability of the mortars prepared with these aggregates. This interaction between aggregate density and mortar flowability should be considered when designing mixtures to achieve both mechanical performance and desired fresh-state properties.

#### 3.2.4. Test Results of Hardened Cement Composite

*Flexural strength after 14 and 28 days*

**Figure 14:**
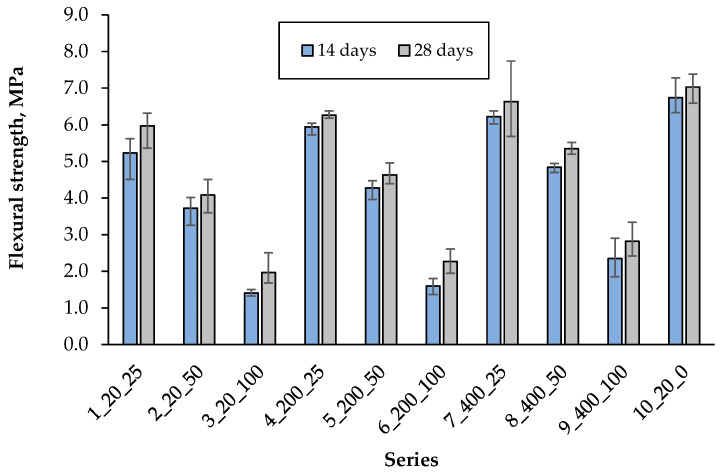
Flexural strength after 14 and 28 days.

The flexural strength was determined after 14 and 28 days of curing, respectively, as an average of 3 samples in each series and the results are shown in Figure 14.

The highest flexural strength after 14 days was recorded in the control series and amounted to 6.74 MPa. With the increase in the hardening temperature of the artificial aggregate from 20 °C to 400 °C, an increase in the composite bending strength was observed by 19%, 30% and 67%, depending on the content of artificial aggregate equal to 25%, 50% and 100%, respectively. Aggregate hardening at 400 °C therefore has the most beneficial effect on the composite’s bending strength. The lowest strength was obtained in the presence of an aggregate cured at 20 °C, which results from the low compressive strength of this aggregate. An increase in the content of AAs from 25% to 100% in the composite resulted in a decrease in flexural strength by about 70%. The greatest increase in strength after 28 days compared to 14 days was observed in the series with 100% content of artificial aggregate. The most favourable strength was obtained in series 7, containing 25% aggregate cured at 400 °C and it was lower than the control series by only 6%, which indicates good adhesion of the porous AA’s surface to the cement matrix.

*Compressive strength after 14 and 28 days*

**Figure 15:**
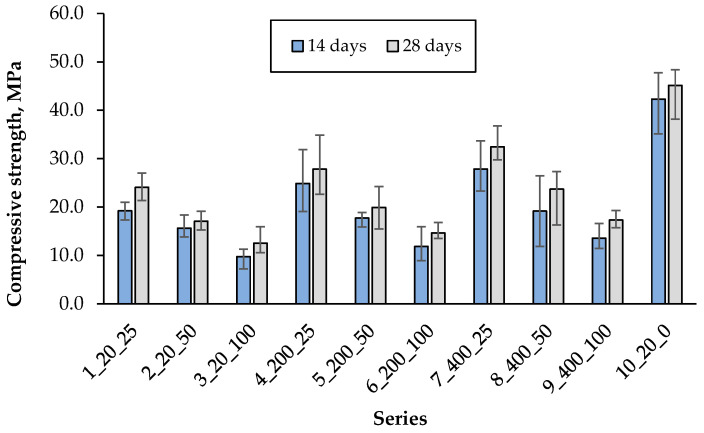
Compressive strength after 14 and 28 days.

Compressive strength was determined after 14 and 28 days of curing, based on the average of six specimens per series. The results are presented in Figure 15.

**Figure 16:**
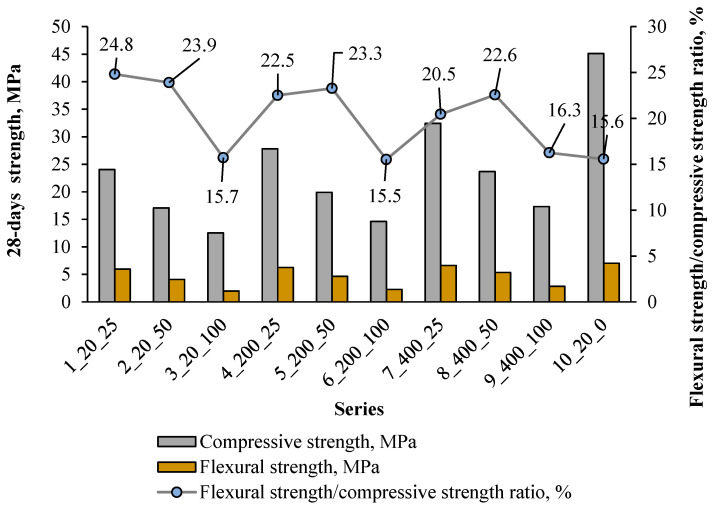
Average compressive strength and flexural strength results after 28 days with flexural/compressive strength ratio.

**Figure 17:**
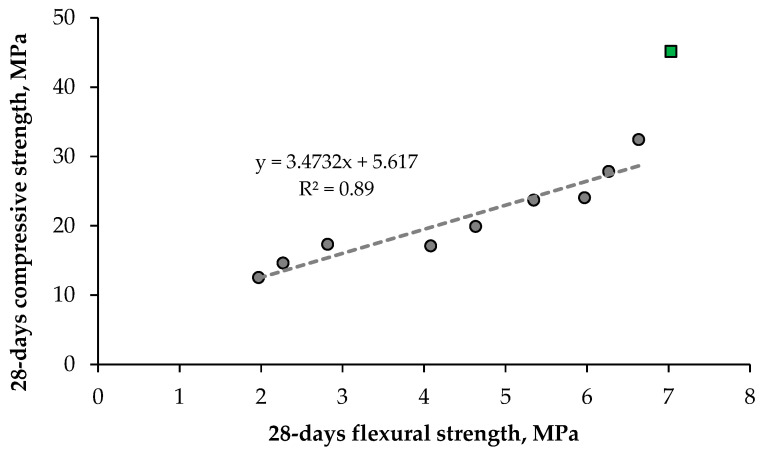
Correlation between compressive strength and flexural strength after 28 days. The green square means a series without artificial aggregates content.

Figure 16 and Figure 17 show the relationship between compressive and flexural strength in individual series.

Figure 16 shows that the compressive strength of the composite with artificial aggregate is significantly lower compared to the control series, even about 3 times lower at 100% AAs content. The flexural strength is about 15.5–24.8% of the compressive strength in the experiment, which is higher than in the case of ordinary. As can be seen from Figure 17, there is a clear linear correlation between compressive and flexural strength in series 1–9. cement composites.

In civil engineering, cement composite is mostly used under compression loading configurations since its compressive strength is much larger than its tensile and/or flexural strengths. Generally, the tensile and flexural strengths of concrete are of the order of 10–15% of the compressive strength [52]. Furthermore, the compressive strength is often considered a marker of the composite quality because it is directly related to the structure of the hydrated cement paste [53]. For these reasons, compressive strength is commonly employed as the primary criterion for assessing the load-bearing capacity and serviceability of concrete structures [54]. In the tested experiment, the highest compressive strength was recorded in the control series (10), which also corresponds to the best bending strength. In the presence of artificial aggregate, a decrease in this strength was noted by 46%, 38% and 28% when using only 25% of artificial aggregate cured at 20 °C, 200 °C and 400 °C, respectively, compared to the control series. Increasing the content of artificial aggregate from 25% to 100% caused a decrease in compressive strength by about 46–47%, regardless of its curing temperature. The test results show that the highest compressive strength with artificial aggregate was obtained with 25% of its addition after curing at 400 °C, which is also confirmed by the flexural strength results.

*Water absorption*

**Figure 18:**
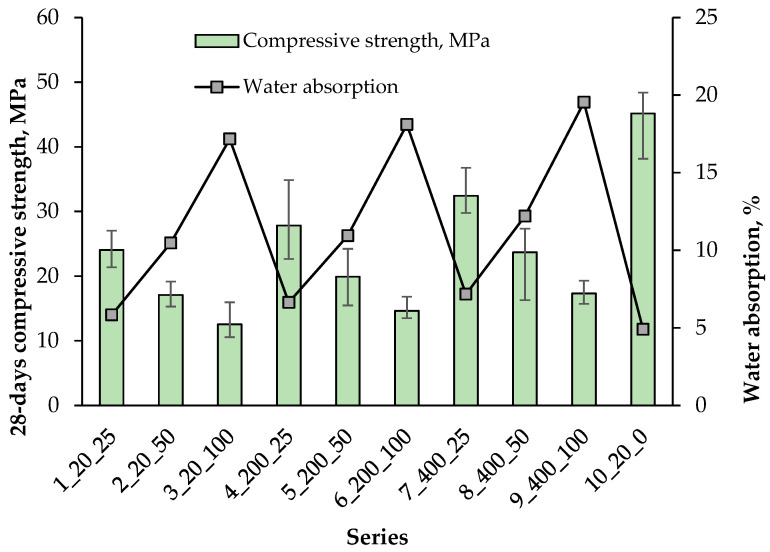
Relationship of 28-day compressive strength and water absorption.

The water absorption of the composite was determined as the average of 3 samples in each series, and the results are presented in Figure 18 along with the compressive strength results after 28 days.

The highest water absorption in the range of 17.2–19.5% was noted in the series containing 100% artificial aggregate. In the presence of 25% AAs, the water absorption of the composite ranged from 5.8 to 7.2%, which indicates a clear dependence of the composite water absorption on the content of artificial aggregate. A gradual increase in water absorption was also observed, on average by about 0.7% and 1.8% with the increase in the hardening temperature of the artificial aggregate from 20 °C to 200 °C and 400 °C, respectively. The greatest increases in water absorption were observed in the series with 100% AAs content. This was directly related to the high water absorption of the aggregates themselves, heated especially at 400 °C. Such aggregates absorbed moisture from the composite, creating a porous structure of the surrounding cement matrix. The control series containing 100% natural aggregate was characterised by the lowest water absorption of 4.9%.

**Figure 19:**
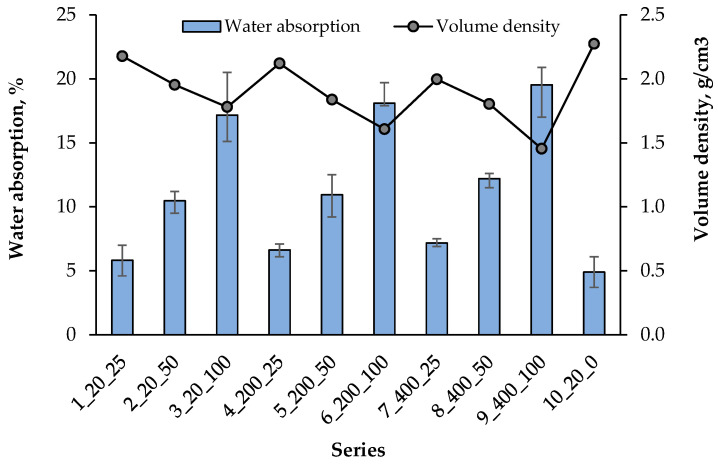
Relationship of water absorption and volume density.

Figure 18 shows the relationship between the compressive strength of the composite after 28 days and its water absorption. As expected, there was a clear relationship according to which the increase in the water absorption of the composite decreased its compressive strength. This is mainly due to the increased porosity of the artificial aggregates used, which reduces the density of the structure and decreases the ability to transfer stress. This relationship is also confirmed by other researchers [55]. The bulk density of the composite was determined as the average of 3 samples in each series, and the results are presented in Figure 19 together with the water absorption results.

*Volume density*

A clear relationship was observed that with the increase in the water absorption of the composite, its density generally decreased (Figure 19). With the increase in the content of artificial aggregate, the density decreased by 18%, 24% and 27%, depending on the temperature of the aggregate heating, 20 °C, 200 °C and 400 °C, respectively. The greatest reduction in the composite density was associated with the use of 100% artificial aggregate hardened at 400 °C (series 9). Only in the series containing 25% of artificial aggregate (1, 4, 7) and in the control (10), the bulk density was higher than 2.0 g/cm^3^. In the remaining ones, it was lower, reaching even 1.45 g/cm^3^ in series 9.

## 4. SEM Images Analysis

**Figure 20:**
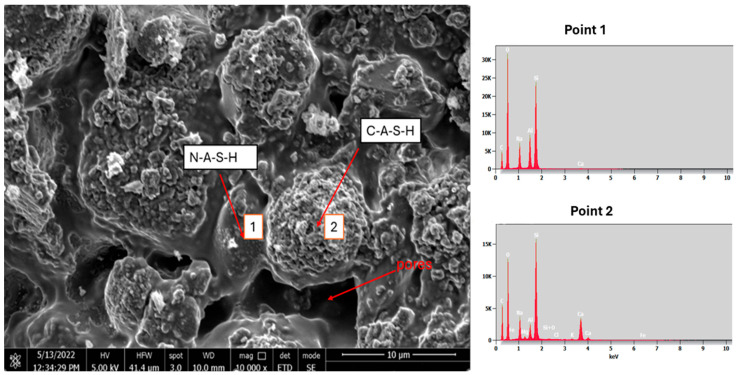
SEM and EDS images of the external surface morphology of artificial aggregate mag. × 10,000.

**Figure 21:**
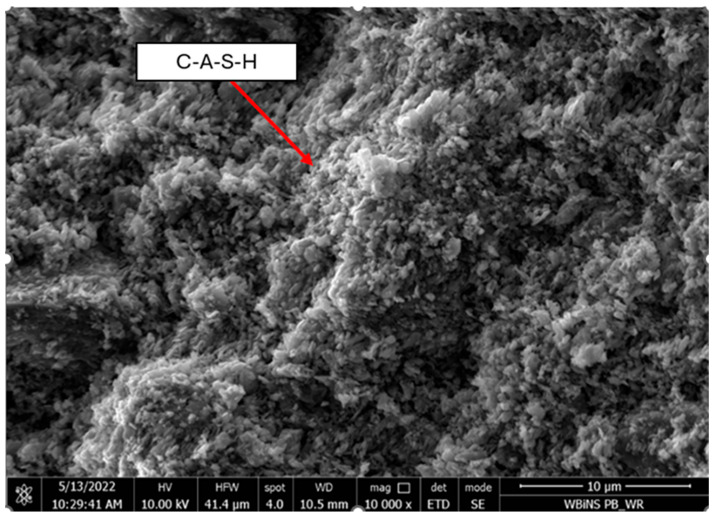
SEM image of the internal structure of an artificial aggregate, mag. × 10,000.

Figure 20 and Figure 21 show scanning electron micrographs of the artificial aggregate at 10,000× magnification with EDS.

The external surface (Figure 20) displays two characteristic amorphous gel phases produced during the alkali activation. Smooth, continuous regions between the granular particles correspond to sodium-aluminosilicate hydrate (N-A-S-H) gel, whereas brighter, irregularly shaped domains (Figure 20 and Figure 21) are attributed to calcium-(alumino)-silicate hydrate (C-A-S-H) gel, formed through the reaction of recycled mortar with the aqueous sodium-silicate solution, consistent with previous findings [56]. In both figures, pores and microcracks are evident; these voids result from the withdrawal of mixing water during the early setting of the fresh mortar and may influence the final porosity and mechanical performance of the aggregate [57].

## 5. Environmental Impact of Aggregate and Composite Manufacturing

The carbon footprint of artificial aggregate production was estimated using a life-cycle assessment (LCA) approach in accordance with the principles of ISO 14040/44 [58,59]. A cradle-to-gate system boundary was adopted: the calculations include the extraction and processing of raw materials (e.g., recycled cement mortar, sediment, incineration ashes, metakaolin), the preparation of the 10 M NaOH solution, the supply of process water, and the electricity demand for the granulation step. For each input, emission factors expressed as kg CO_2_-equivalent per tonne of material or per unit of energy were taken from published literature and national databases (values are shown in Table 11 and Table 12). The total carbon footprint for each aggregate composition was then obtained by multiplying the mass of each component by its specific emission factor and summing the contributions of all processes.

It should be noted that the calculations did not take into account the CO_2_ absorption capacity of the individual waste materials, which could significantly reduce their carbon footprint. It is also worth emphasising that fine mortar, sediment, and incineration ash are difficult-to-manage wastes, which substantially increases the value of the proposed solution; however, this aspect is intangible. According to Table 11, composition C—characterised by the highest content of DFR and lower amounts of sediment and incineration ash—exhibits the lowest emissions. The highest carbon footprint among the aggregate compositions is associated with the 10 M NaOH solution, indicating that future studies should consider reducing its concentration. It should be stressed that these are preliminary findings, as further research on CO_2_ sequestration in artificial aggregate is necessary. Table 12 presents the emission results for the cementitious composite prepared with artificial aggregate of composition B, used in proportions ranging from 0% to 100% by volume of the coarse aggregate fraction (2–8 mm).

Compared to composites with natural aggregate, CO_2_ emissions for composites containing artificial aggregate derived from waste are higher, ranging from 20% to 60%, depending on the content of artificial aggregates (AAs). The relatively high carbon footprint in the current state results, among other factors, from energy-intensive processes, such as drying and granulation, which are not needed in natural resources. The use of renewable energy sources could significantly reduce this footprint. It should be remembered that the natural aggregate resources are being depleted, while the amount of waste continues to increase. For this reason, the use of artificial aggregates produced from waste will become a necessity, and through further improvements in production technologies, their carbon footprint could be reduced. Moreover, the artificial aggregates proposed in the article require thermal treatment at only 400 °C. In contrast, the production of materials such as expanded clay or foam glass, which exhibit similar strength parameters, involves processing temperatures of around 1100–1200 °C.

Despite the higher CO_2_ emissions associated with the production of alkali-activated artificial aggregates, their application aligns with sustainable development strategies by reducing waste volumes, decreasing the consumption of natural resources, and allowing for further optimisation of the production process. From a systems and life-cycle perspective, these aggregates may represent a competitive and more environmentally responsible alternative to natural materials, particularly within the framework of a circular economy.

## 6. Conclusions

The conducted research aimed to explore the possibilities of waste management through the utilisation of ash, sludge, and mortar for the production of artificial aggregate. To this end, the most advantageous proportions of waste components, the type of activator, and the curing temperature of the aggregate were determined. Subsequently, the influence of the artificial aggregate on the properties of cementitious composites was assessed.

The obtained results led to the following conclusions:

- It is possible to produce artificial aggregate containing up to 70% waste materials; however, the use of an alkaline activator is necessary.
- The highest compressive strength of the aggregate was achieved at a 50% content of recycled cement mortar and a curing temperature of 400 °C. Thermal curing plays a crucial role in the polymerization process; however, it also increases the aggregate porosity due to the combustion (or thermal decomposition) of fine particles, particularly those originating from incineration ash.
- The presence of artificial aggregates significantly reduces the workability of fresh composites due to their high water absorption; therefore, the use of plasticizers should be considered when their content exceeds 50%.
- The best compressive and flexural strength results of composites with artificial aggregates were obtained in Series 7, containing 25% Aas, cured at 400 °C.
- At artificial aggregate contents above 25%, the composite’s density falls below 2.0 g/cm^3^, classifying it as lightweight; however, this is accompanied by increasing water absorption.
- The produced artificial aggregates can replace natural aggregates up to 25%, representing an important form of waste valorisation and aligning with circular economy principles.

In summary, this study confirms that artificial aggregates can be manufactured from three waste streams through cold-bonding, and that their incorporation up to 25% by volume enables the production of lightweight cementitious composites without critical degradation of mechanical performance. Although the produced aggregates are still characterised by low individual grain strength and high water absorption, this performance level is already sufficient for non-load-bearing and lightweight construction applications, where weight reduction and waste valorisation are more important than strength maximisation. It must be emphasised that the carbon footprint values reported here are conservative, as potential post-production CO_2_ uptake by waste-derived aggregates was not included in the LCA system boundary. Therefore, future work should focus on (i) quantifying the long-term carbon sequestration potential of these aggregates, (ii) reducing the embodied emissions of alkali activators, and (iii) prototype-scale validation in lightweight construction components. Collectively, these steps are essential to position waste-derived artificial aggregates as a technically credible and environmentally competitive circular-economy alternative to natural aggregates.

## Figures and Tables

**Table 1:** Cement CEM II B-V 32.5R specifications.

Parameter	Average Value of the Parameter
Blaine’s specific surface area, cm^2^/g	3522.0
Initial setting time, min	298.0
Final setting time, min	397.0
Compressive strength after 2 days, MPa	17.20
Compressive strength after 28 days, MPa	47.95
Specific density, g/cm^3^	2.81
Water demand, %	28.7
Sulfate content (as SO_3_), %	2.64
Chloride content (as Cl), %	0.063
Alkali content (as Na_2_O), %	1.3

**Table 2:** The chemical composition of waste raw materials and metakaolin used for the production of artificial aggregate.

Component	Municipal Waste Incineration Ash (MWIA)	Sediment from the Bottom of a Water Reservoir (SBWR)	Recycled Cement Mortar (RCM)	Metakaolin (MK)
	(%)			
Na_2_O	4.65	-	-	0.03
MgO	6.57	1.15	1.59	0.02
Al_2_O_3_	0.53	9.36	2.85	50.24
SiO_2_	1.48	65.53	34.40	41.90
P_2_O_5_	-	0.32	-	0.10
SO_3_	6.99	0.46	0.85	0.04
Cl	1.04	-	0.03	-
K_2_O	5.45	2.82	1.08	0.25
CaO	48.04	5.31	39.39	0.34
TiO_2_	0.30	0.80	0.19	1.12
Cr_2_O_3_	0.01	0.02	-	0.02
MnO	0.05	0.14	0.06	0.003
Fe_2_O_3_	0.48	5.14	2.13	1.95
CuO	0.04	-	-	0.002
ZnO	1.04	0.02	0.04	0.01
LOI	23.33	8.78	17.34	0.63

**Table 3:** Content of selected components of RCM.

**RCM**	**Components of the RCM, % of Mass**				
	**Bound Water**			**Ca(OH)_2_**	**CaCO_3_**
	**H_I_**	**H_CH_**	**Σ**		
	2.28	1.39	3.67	5.71	27.83

**Table 4:** The hardening temperature (X_1_) and RCM content (X_2_) for aggregates series.

X_1_—Hardening Temperature of Aggregates [°C]	X_2_—RCM Content in the Ingredients Mass [%]
20	40
200	50
400	60

**Table 5:** Formulation of Artificial Aggregate Mixtures.

Component	Amount, %		
	Composition A	Composition B	Composition C
RCM	40	50	60
MWIA	17.5	10	10
MK	17.5	10	10
SBWR	5	10	0
NaOH 10 M (16% + 4% water)	20	20	20

**Table 6:** Experimental design matrix presenting the combinations of temperature (X_1_) and X_2_ levels used in each series. X_2_ categories (A, B, C) correspond to three variants of the proportion of the 2–8 mm fraction in the artificial aggregate.

Series	Variables	
	X_1_, °C	X_2_, [%]
1	20	A
2	20	B
3	20	C
4	200	A
5	200	B
6	200	C
7	400	A
8	400	B
9	400	C

**Table 7:** Average properties of artificial aggregates depending on composition and curing temperature.

Series	Composition/Fraction, mm		Hardening Temperature, °C	Bulk Density in a Loose State, g/cm^3^	Bulk Density in a Compacted State, g/cm^3^	Volume Density, g/cm^3^	Water Absorption, % Mass	Compressive Strength, MPa
1	A	2–8	20 °C	0.86	1.01	1.98	10.3	0.40
		8–32		0.74	0.84	2.05	7.2	
2	B	2–8		0.95	1.06	2.10	8.9	0.57
		8–32		0.77	0.86	1.90	9.3	
3	C	2–8		0.96	1.07	2.04	9.6	0.51
		8–32		0.77	0.86	1.99	8.0	
4	A	2–8	200 °C	0.82	0.98	1.83	15.5	0.43
		8–32		0.68	0.80	1.61	19.9	
5	B	2–8		0.95	1.05	1.97	11.6	0.77
		8–32		0.76	0.91	1.89	9.9	
6	C	2–8		0.99	1.10	1.89	14.2	0.41
		8–32		0.81	0.92	2.04	7.7	
7	A	2–8	400 °C	0.85	0.98	1.81	16.2	0.64
		8–32		0.68	0.78	1.70	14.5	
8	B	2–8		0.87	1.01	1.83	15.8	0.93
		8–32		0.77	0.84	1.71	14.7	
9	C	2–8		0.95	1.06	1.94	12.3	0.58
		8–32		0.73	0.86	1.77	13.3	

**Table 8:** Factor levels for temperature (X_1_) and the proportion of the 2–8 mm fraction (X_2_).

X_1_—Temperature of Aggregate Hardening, °C	X_2_—Proportion of 2–8 mm Fraction of Artificial Aggregate, %
20	25
200	50
400	100

**Table 9:** The full Experimental series coding and corresponding factor levels for aggregate hardening temperature (X_1_) and proportion of 2–8 mm fraction (X_2_) in cement composites.

Series	Variables	
	X_1_, °C	X_2_, %
1_20_25	20	25
2_20_50	20	50
3_20_100	20	100
4_200_25	200	25
5_200_50	200	50
6_200_100	200	100
7_400_25	400	25
8_400_50	400	50
9_400_100	400	100
10_20_0	20	0

**Table 10:** Composite’s mixture depending on the percentage content of artificial aggregate.

Composition	Artificial Aggregate Content			
	0%	25%	50%	100%
CEM II B-V 32.5R, [kg/m^3^]	320	320	320	320
Water [kg/m^3^]	160	160	160	160
*w*/*c*	0.50	0.50	0.50	0.50
Sand 0–2 mm [kg/m^3^]	878.6	878.6	878.6	878.6
Gravel aggregate 2–8 mm [kg/m^3^]	1073.8	805.4	536.9	0
Artificial aggregate 2–8 mm [kg/m^3^]	0	152	303.9	607.8

**Table 11:** CO_2_ Emissions for aggregates.

Component	Carbon Footprint, kg CO_2_ e/t	Artificial Aggregate Composition		
		A	B	C
RCM	0.598 [60]	0.24	0.30	0.36
SBWR	242.02 [61]	42.35	24.20	24.20
MWIA	400 ^(1)^ [62]	70.00	40.00	40.00
MK	330 [63]	16.50	33.00	0.00
NaOH, 10 M	332 ^(2)^ (830 for NaOH) [64]	53.12	53.12	53.12
water	0	0.00	0.00	0.00
granulation	10.5 ^(3)^	10.50	10.50	10.50
**Total, kg CO_2_/t for artificial aggregate**		**192.71**	**161.12**	**128.18**

^(1)^—The calorific value was assumed to be 10 GJ/t (emission: 10 GJ/t × 40 kg CO_2_/GJ = 400 kg CO_2_/t); ^(2)^—calculated for a 10 M NaOH solution; ^(3)^—Energy consumption for a medium-sized granulator was assumed to be 15 kWh/t; the carbon footprint of electricity in Poland is 0.7 kg CO_2_/kWh; thus, the carbon footprint of the granulation process is: 15 kWh/t × 0.7 kg CO_2_/kWh = 10.5 kg CO_2_/t.

**Table 12:** CO_2_ Emissions for cement composites.

Composition	Carbon Footprint (kg CO_2_ e/t)	Carbon Footprint kg/m^3^			
		0%	25%	50%	100%
CEM II B-V 32.5R, kg/m^3^	473.0 ^(1)^	151.36	151.36	151.36	151.36
Water, kg/m^3^	0	0	0	0	0
Sand 0–2 mm, kg/m^3^	6.0 ^(2)^	5.2716	5.2716	5.2716	5.2716
Gravel aggregate 2–8 mm, kg/m^3^	9.0 ^(2)^	9.6642	7.2486	4.8321	0
Artificial aggregate 2–8 mm, kg/m^3^	161.12	0.000	24.490	48.965	97.929
**Total, kg CO_2_ e/m^3^ of composite**		**159.9**	**188.9**	**210.9**	**255.1**

^(1)^—www.cemex.pl, accessed on 6 November 2025; ^(2)^—https://www.climatiq.io/data, accessed on 6 November 2025.

## Data Availability

The data presented in this study are available on request from the corresponding author. The data are not publicly available due to commercial sensitivity and potential disclosure of proprietary research know-how.
